# Isolation, characterization and screening of actinomycetes associated with fijian ant–plant symbioses

**DOI:** 10.1099/mic.0.001410

**Published:** 2023-11-08

**Authors:** Umar Diarra, Tamara Osborne-Naikatini

**Affiliations:** ^1^​ School of Agriculture, Geography, Environment, Ocean and Natural Sciences, The University of the South Pacific, Laucala Campus, Suva, Fiji

**Keywords:** actinomycetes, ant–plants, bioactive, Hymenoptera, symbiosis, *Squamellaria*, Actinomycetota

## Abstract

In the search for novel therapeutics to combat the ongoing antimicrobial resistance crisis, scientists are turning to underexplored environments. Defensive mutualisms between hymenopteran insects and actinomycetes represent important reservoirs for bioactive compounds. In this study, we examined the association between actinomycetes and *Squamellaria* ant-plants spanning three different ant and plant species combinations (*Squamellaria imberbis-Philidris nagasau, Squamellaria tenuiflora- Technomyrmex vitiensis*, and *Squamellaria tenuiflora-Tetramorium insolens*). Eight *Squamellaria* plants were sampled including four containing *T. vitiensis,* three containing *P. nagasau,* and a single plant containing *T. insolens*. A total of 47 actinomycetes were obtained from the sampled material, with 5, 16, and 26 isolates originating from cuticle, tissue, and nest samples, respectively. Cross-streaking tests showed that 12 out of 47 isolates inhibited bacterial pathogens. The most frequently inhibited pathogens in the cross-streaking tests were *S. aureus* and *E. coli* while *S. enterica* was the least inhibited. Among the three primary screening media used, ISP2 agar was the most suitable for secondary metabolism as more isolates exhibited antibacterial activity when grown on the medium. TFS2010 and TFS2003, which matched to *Streptomyces gramineus* (>99% similarity), were the most bioactive isolates in cross-streaking tests. TFS2010 displayed the strong antibacterial on Nutrient agar, Mueller Hinton agar, and ISP2 agar while TFS2003 only exhibited strong antibacterial activity on Nutrient agar. Furthermore, a difference in potency of extracts based on batch culture medium was noted in TFS2010**.** DNA was extracted from 19 isolates and followed by 16SrRNA gene sequencing. Analysis of sequence data revealed the presence of six genera, including *Amycolatopsis *, *Asanoa *, *Jiangella *, *Nocardia *, *Nocardiopsis *, and *Streptomyces *, with the latter being the most abundant taxon. Among these, three isolates (PNS3002, PNS3005, and TFS3001) are likely to represent new species while one (TFS2015) is likely to be a member of a novel genus. Our work represents the first attempt to study actinomycetes from *Squamellaria*-ant mutualisms.

## Introduction

The current global health crisis necessitates the discovery of novel antibiotics and drugs for the prevention and treatment of emerging deadly human pathogens. Despite the gradual shift from natural to synthetic drug discovery in recent decades, natural products remain a top source of novel drugs and bioactive compounds. According to Newman and Cragg [1], drugs sourced from nature account for about 23 % of 1881 new approved drugs discovered between January 1981 and September 2019. Furthermore, 18.9 % of the drugs are semisynthetic derivatives of natural products while 25.7 % are synthetic drugs modelled based on existing natural products. With vaccines accounting for a further 7.5 %, only 24.6 % of new approved drugs are truly synthetic. Natural products have been isolated from a wide array of organisms, including plants, vertebrates and invertebrates. Nonetheless, microbes are arguably the most important source of natural products, accountable for about 30 000 natural products in use today [2].

Among micro-organisms, bacteria of phylum Actinomycetota, formerly known as Actinobacteria, are particularly well known as producers of bioactive compounds. Members of Class Actinomycetia, also known as actinomycetes, are filamentous, Gram-positive bacteria that are widely distributed in marine and terrestrial environments. About two-thirds of all naturally derived antibiotics have been obtained from actinomycetes, with members of the genus *Streptomyces* accounting for up to 70 % production [2, 3]. Furthermore, rare actinomycetes, which are non-streptomycetes belonging to genera such as *Micromonospora, Pseudonocardia, Nocardiopsis, Nocardia, Actinomadura*, *Amycolatopsis* etc., account for the remaining 30 % of actinomycete-derived antibiotics [4]. Despite the prominence of actinomycetes in antibiotic discovery, intensive screening of common actinomycetes (especially *Streptomyces* spp.) coupled with limitations of conventional isolation and screening methods have led to a significant decline in the rate of discovery of novel natural products from actinomycetes in past decades [3]. Among the common strategies employed in actinomycetes research today, is the exploration of previously underexplored and unexplored environments.

Symbiotic associations between organisms represent a unique and underexplored environment for actinomycetes research. Perhaps the best studied symbiosis in this regard is the fungus-farming mutualism of attine ants. Attine ants, ranging from leafcutters of the genera *Atta* and *Acromyrmex* to more primitive genera such as *Cyphomyrmex* and *Cyatta*, are known to cultivate basidiomycete fungi as food. However, attine ant fungal gardens are prone to infections by pathogenic fungi of the genus *Escovopis,* which coevolved alongside the ants and their food source. In response, the ants have developed behavioural adaptations, such as fungus garden grooming, as well as a symbiotic association with actinomycetes of the genus *Pseudonocardia *, to maintain nest hygiene and control garden parasites. Several novel compounds have been reported from attine ant-associated *Pseudonocardia *, including Pseudonocardone A, B and C [5], 9-methoxyrebeccamycin [6], Dentigerumycin [7], Gerumycin A, B and C [8], Nystatin-like antifungals [9–11], and the atypical antifungal polyene Selvamicin [12].

Ant–plant symbiosis describes a form mutualism involving 113 species of ants and about 684 species of vascular plants [13]. In ant–plant mutualisms, the ants are usually involved in dispersing the seeds of the host plant (Myrmecochory), protection of the growing plant, and in some cases, provisioning of essential nutrients. On the other hand, ant–plants, also known as myrmecophytes, have evolved specialized chambers known as domatia to house ants. In addition, some species of myrmecophytes are known to produce extrafloral nectaries, which help to supplement the diets of the ants [14]. Actinomycetes have been reported from ant–plant symbioses such as the *Allomerus* sp. and *Hirtella* sp. symbiosis [15], *Allomerus octoarticulatus* and *Cordia* sp. symbiosis [15], *Crematogaster margaritae* and *Keetia hispida* symbiosis [16], *Pseudomyrmex penetrator* and *Tachigali* sp. symbiosis [16], *Petalomyrmex phylax* and *Leonardoxa africana* symbiosis [16], and *Tetraponera penzigi* and *Vachellia drepanolobium* symbiosis [15]. Additionally, the novel antimicrobial compounds; filipins [17], formicamycins [18] and kyamicin [19] were isolated from actinomycetes associated with ant–plant systems.

The plant family Hydnophytinae (Rubiaceae, Psychotriae) is the largest group of ant–plants, comprising of five genera (*Hydnophytum*, *Myrmecodia*, *Anthorrhiza*, *Myrmephytum* and *Squamellaria*) and approximately 117 species of epiphytic ant–plants distributed across South-east Asia and Oceania [20, 21]. The genus *Squamellaria* (Family Rubiacea) includes 12 species of myrmecophytes, nine of which are native to Fiji, two to Solomon Islands and one to Vanuatu [22]. Of the nine species endemic to Fiji, six including *S. grayi*, *S. huxleyana*, *S. imberbis*, *S. major*, *S. thekii* and *S. wilsonii*, are known to be involved in an obligate mutualism with the endemic ant *Philidris nagasau* (Formicidae, Dolichoderinae) [23]. The remaining three *Squamellaria* species of Fiji have been shown to associate with a few native ant species including *Pheidole knowlesi* and *Technomyrmex vitiensis*, nonetheless facultatively. The obligate symbiosis between *Squamellaria* spp. and *P. nagasau* is unique among ant–plant symbioses as it is a farming mutualism (ants cultivate *Squamellaria*) just like attine ant agriculture. Furthermore, the mutualism is characterized by a high level of dependence of both organisms on the mutualism, leading some authors to term it as obligate dependency [24]. Ant-exclusion experiments conducted by Chomicki and Rennner [25] revealed that obligate species of *Squamellaria* had significantly higher mortality than their facultative counterparts, suggesting that *P. nagasau* ants may provide protection from nest parasites. Here, we demonstrate, using culture-dependent techniques, that diverse bioactive actinomycetes are associated with both obligate and facultative *Squamellaria* and their respective ant species.

## Methodology

### Sampling

*Squamellaria* plants were collected from the Islands of Viti Levu and Vanua Levu, Fiji between November 2021 and July 2022. Two species of *Squamellaria, S. imberbis* and *S. tenuiflora,* were sampled in this study. *S. imberbis* is native to Fiji and grows exclusively on the Island of Vanua Levu [23]. Like all obligate *Squamellaria* in Fiji, *S. imberbis* is associated with *Philidris nagasau*. For this study, three samples of *S. imberbis* were collected near Waisali Rainforest Reserve in Vanua Levu (16 °37'46.8"S 179 °12'14.8"E). *S. tenuiflora,* on the other hand, occurs on Viti Levu and Ovalau, and domatia of the species are known to be inhabited by native ant species [23]. In this study, two ant species, *Technomyrmex vitiensis* and *Tetramorium insolens*, were found inhabiting *S. tenuiflora* samples, which were collected from Colo-i-Suva Forest Reserve (18 ° 3’ 34.92’ S, 178 ° 27’ 29.88’ E) in Viti Levu.

Whole *Squamellaria* plants containing ant colonies were collected by detaching roots from tree stems. The plant samples were placed in sterile bags and immediately transported to the laboratory for processing.

**Fig. 1.**
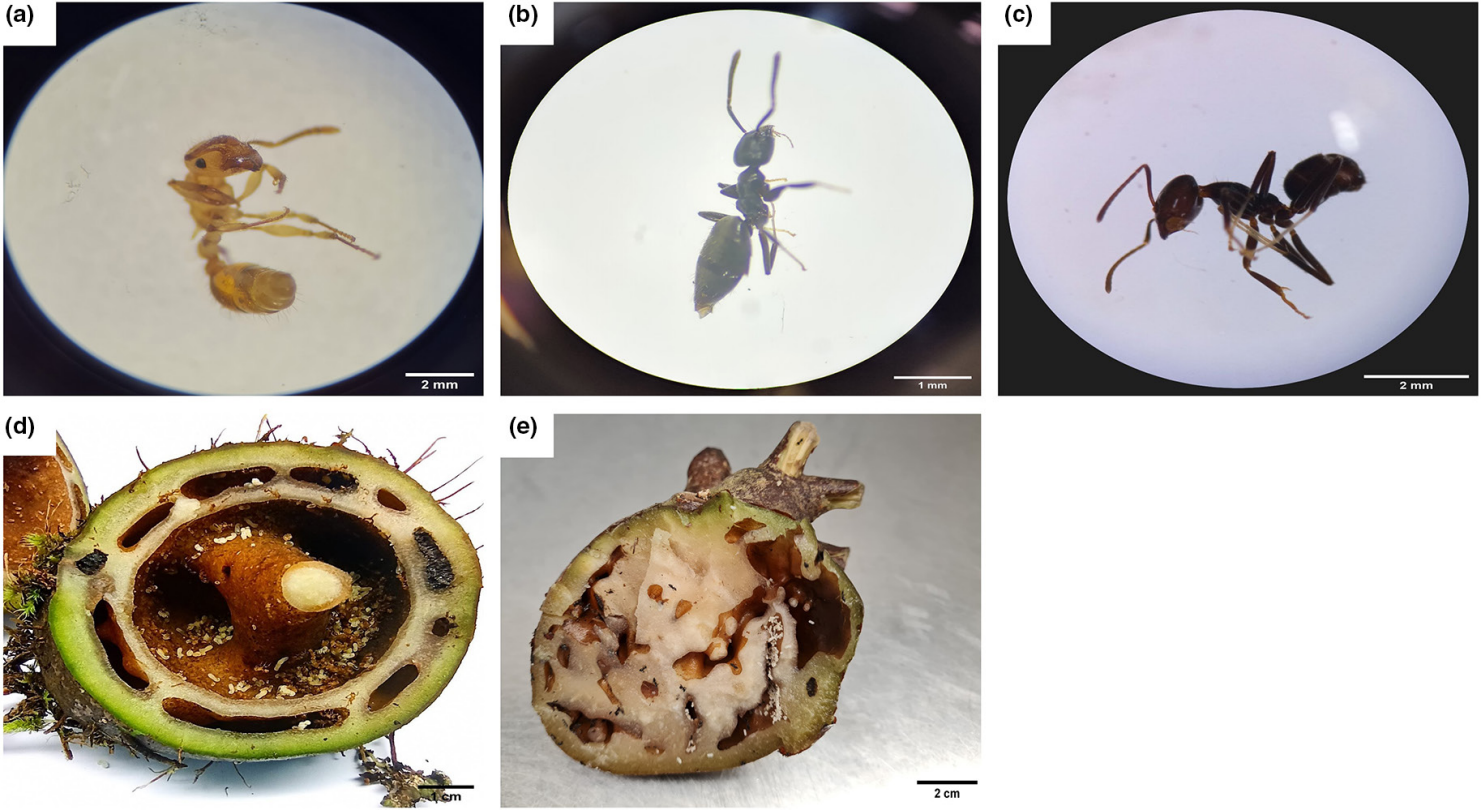
Ant-plants and associated ant species. (**a**) *Tetramorium insolens* viewed under a dissecting microscope. (**b**) *Technomyrmex vitiensis* viewed under a dissecting microscope. (**c**) *Philidris nagasau* viewed under a dissecting microscope. (**d**) Dissected *Squamellaria tenuiflora* showing *T. insolens* nest. (**e**) Dissected *Squamellaria tenuiflora* showing *T. vitiensis* nest. All pictures were taken by the principal author. Image not available for *Squamellaria imberbis*.

Once at the lab, *Squamellaria* plants were dissected aseptically to expose the domatia (Fig. 1). Twenty to thirty worker ants were randomly collected from the domatia of each plant using sterile forceps and placed in sterile vials. Three to five workers of each ant species were used for identification. Selected individuals were euthanized by immersion in 70 % alcohol and subsequently studied under a dissecting microscope. Species-level identification was done based on morphological characteristics, such as colouration, size and shape of features. For further validation, samples were sent to specialists at the Ministry for Primary Industries, New Zealand.

### Isolation of ant-associated actinomycetes

In an effort to optimize the recovery of actinomycetes from hymenopteran specimens, we employed a diverse range of isolation media. Specifically, three distinct types of isolation media, namely Humic acid Vitamin agar [26], Chitin agar [16, 27] and Gause’s synthetic agar No. 1 [28], were utilized for the isolation of actinomycetes from tissue and cuticle samples. All isolation media were supplemented with 50 mg/L cycloheximide and 20 mg/L nalidixic acid to suppress the growth of fungi and Gram-negative bacteria, respectively.

Prior to isolation, ant samples were refrigerated at 4 °C for 3–4 h to allow for easy manipulation. For each ant species, about 1 g of sample was used for actinomycete isolation from the cuticle and tissue. To isolate actinomycetes from the cuticle, approximately 1 g of the sample was placed in a sterile Eppendorf tube, rinsed twice with sterile water to remove the surface soil and adherents, and immersed in 1 ml sterile water with vortexing for 30 s. Finally, 100 µl of the resulting suspension was spread on each of the different isolation media while maintaining three replicates.

After isolation of actinomycetes from the cuticle, the previously washed samples were surface sterilized by immersing in 70 % alcohol for 60 s, followed by washing three times with sterile distilled water. Each sample was then macerated in a sterile mortar and pestle, followed by immersion in 1.0 ml sterile water and vortexing for 20 s [27]. Finally, 100 µl of this tissue suspension was spread on each of the different isolation media while maintaining three replicates.

### Isolation of *Squamellaria*-associated actinomycetes

From each dissected *Squamellaria* plant, about 10 g of the composite sample consisting of nest debris, tuber tissue, and smooth and warty wall tissues (present in obligate species) was obtained. The nest sample was suspended in 90 ml of sterile water and vortexed thoroughly. A 100 µl aliquot of the resulting suspension was then spread on isolation media while maintaining three replicates. Two isolation media, Humic acid Vitamin agar (HVA) and Actinomycetes Isolation agar (AIA) (HiMedia), supplemented with 50 mg l^−1^ cycloheximide and 20 mg l^−1^ nalidixic acid, were used for the isolation of actinomycetes from *Squamellaria* samples. Spread plates were incubated for 3–4 weeks at 28 °C.

### Purification and morphological description of actinomycetes

Following enumeration, the morphologies (e.g. size, form, coloration, texture, margin, elevation) of actinomycete colonies growing on isolation plates were recorded and isolates were assigned unique alphanumeric codes based on the samples and host colonies they were derived from. Individual actinomycete colonies were then purified by quadrant streaking on yeast extract-malt extract agar, also known as International streptomyces project 2 agar (ISP2A) (HiMedia), agar plates which were subsequently incubated at 28 °C for 5–7 days.

For long-term preservation of isolates, glycerol stocks were prepared according to the procedure described by El Karkouri *et al*. [29].

### Primary screening of isolates for antimicrobial activity

The antimicrobial activity of actinomycete isolates was determined using the modified cross-streak method [30]. Due to the vital role nutrients play in actinomycete secondary metabolism, three different media; Mueller–Hinton agar (MHA), Nutrient agar (NA) and ISP2A, with varying carbon and nitrogen sources were employed for the cross-streaking tests. Each actinomycete isolate was streaked on the corner of an agar plate followed by incubation for 7–10 days at 28 °C. Following incubation, seven human pathogenic bacteria including *Enterococcus faecalis* ATCC 19433, *Escherichia coli* ATCC 25922, *Pseudomonas aeruginosa* ATCC 25668, *Klebsiella aerogenes *, *Salmonella enterica* NCTC 7836, *Shigella sonnei* ATCC 9290 and *Staphylococcus aureus* ATCC 25923, were streaked perpendicular to the actinomycete. Cross-streaked plates were sealed with parafilm and incubated at 37^o^ C for 24 h. Incubated plates were observed for inhibition zones which were measured to the nearest millimetre using a ruler.

### Preparation of batch cultures and extraction of secondary metabolites

Isolates that displayed significant antimicrobial activity in primary screening tests were selected for batch cultivation and secondary metabolite extraction. Each of the selected isolates was grown in a liquid medium corresponding to the solid medium on which the best bioactivity was recorded during primary screening (e.g. nutrient agar-active isolates were grown in nutrient broth). To prepare batch cultures, each of the isolates was inoculated into 100 ml of sterile broth contained in a 250 ml conical flask. Conical flasks were sealed with cotton plugs and then placed in a shaking incubator set at 28 °C and 150 r.p.m. for 14 days. Following incubation, broths were centrifuged at 10 000 r.p.m. and filtered using a Whatman no.1 filter paper and the resulting supernatant was collected.

Two solvents (Ethyl acetate and water) with relatively different polarities were chosen for secondary metabolite extraction. For ethyl acetate extraction, equal volume of ethyl acetate was added to about 50 ml of supernatant contained in a 250 ml glass bottle. The glass bottle was sealed tightly and shaken for 12 h on a rotary shaker. After shaking, the contents of the bottle were then transferred to a 500 ml separating funnel, shaken vigorously for about 2 min, and left to stand for 1–2 h for the layers to separate. Once two layers were visible, the bottom (aqueous) layer was drained and discarded while the top (organic) layer was carefully transferred into a pre-weighed round-bottom flask and concentrated on a rotary evaporator. After the organic layer had been evaporated, the round bottom flask containing the samples were again weighed. The mass of crude obtained was calculated using the following formula:

Mass of crude=final mass of RBF – initial mass of RBF (2)

The remaining 50 ml of supernatant was placed in a pre-weighed 50 ml centrifuge tube and frozen for 24 h at −80 °C. The frozen sample was then transferred to a Zirbus VaCo 5-II freeze-dryer set at a temperature of −60 °C and pressure of 0.0001 atm. The freeze-drying programme was run for about 72 h and the mass of the resulting dry crude was calculated using equation 2 above.

In preparation for broth microdilution tests, ethyl acetate and aqueous extracts were diluted using a sterile solution of 5 % Dimethyl sulfoxide (DMSO) to obtain a stock concentration of 50 mg ml^−1^ for each extract. The volume of diluent needed to dilute each extract was calculated as follows:

Volume of DMSO (ml) required=mass of crude (mg)/50 mg ml^−1^ (3)

### Determination of minimum inhibitory concentration

The minimum inhibitory concentration (MIC) is defined as the lowest concentration of an antimicrobial required to inhibit the growth of a test micro-organism. In this study, the MICs of actinomycete crude extracts were determined using the broth microdilution method as described by Wiegand *et al*. [31].

Before use in broth microdilution tests, cultures of the test pathogens had to be standardized as required by CLSI guidelines [31, 32]. To achieve this, each pathogen was streaked on a MHA plate, which was subsequently incubated at 37 °C for 24 h. After incubation, one–two colonies were aseptically transferred from the culture plate to a 10 ml test tube containing 5 ml sterile cation-adjusted Mueller–Hinton broth (MHB). Test tubes were vortexed thoroughly for 10–15 s and visually compared to a 0.5 McFarland solution with the aid of a Wickerham card. If the bacterial suspension appeared more turbid than the 0.5 McFarland solution, more cation-adjusted MHB was added to the suspension and if less turbid, more bacterial culture was added to the suspension. After adjustment to McFarland 0.5 turbidity, the suspension was further diluted 100-fold by adding 200 µl to 19.8 ml of sterile MHB. The resulting solution had a cell density of approximately 1.5×10^6^ c.f.u. ml^−1^ and was ready to be used as inoculum in the broth microdilution assay.

Broth microdilution assays for the ethyl acetate and aqueous crudes were performed in 96-well plates. For each extract, the test concentrations ranged from 0.049 mg ml^−1^ to 25 mg ml^−1^ (well 1–10) while maintaining one growth control (well 11) and one sterility control (well 12). To obtain the test concentrations, 100 µl of 50 mg ml^−1^ crude extract was added to each of the first two wells (1 and 2). This was followed by the addition of 100 µl of sterile MHB in wells 2–11. Twofold serial dilutions of the extract were made by thoroughly mixing the contents of well 2, i.e. 100 µl of crude and 100 µl of MHB, before transferring 100 µl to well 3. This step was repeated for all subsequent wells until well 10 from which 100 µl was removed and discarded. Wells 1–11 were subsequently seeded with 100 µl each of standardized inoculum. Considering the inoculum was contained in a broth and therefore functioned as a diluent, the final cell density in each of the wells was ~7.5×10^5^ c.f.u. ml^−1^, which is recommended by CLSI. Finally, 200 µl of sterile MHB was pipetted into well 12 and the plates were covered and incubated at 37 °C for 24 h. Crude extracts were tested in triplicates and chloramphenicol, in the range 0.000125 to 0.128 mg ml^−1^, was used as a positive control. To ease the differentiation between positive and negative results, resazurin indicator was used according to Elshikh *et al*. [33].

### DNA extraction and sequencing

Genomic DNA of actinomycete isolates was extracted using GenElute Bacterial Genomic Kit according to the manufacturers’ protocol.

The DNA lysates were packaged and shipped on ice to the Massey Genome Centre (Palmerston North, New Zealand) for further analyses. The 16S rRNA gene was amplified in the DNA lysates using PCR. The reaction mix consisted of 1 µl lysate, 10 pmol each of primers 27F and 1492R, 1X EmeraldAmp Max HS PCR Master Mix (Takara), and water to make a total volume of 20 µl. A negative control without template DNA was included. The PCR was performed in a Biometra T1 Thermocycler (Analytika Jena) with a programme of 95 °C for 10 s, followed by 35 cycles of 98 °C for 10 s, 55 °C for 30 s, and 72 °C for 90 s, with a final extension at 72 °C for 5 min and a hold at 10 °C. The PCR products were separated via gel electrophoresis on 1 % (w/v) agarose prepared as previously described. Gels were visualized and then photographed using a UVIDOC HD6 machine (UVITEC Cambridge). Residual dNTPs were dephosphorylated by adding 0.5U Shrimp Alkaline Phosphatase (rSAP, New England BioLab) and unincorporated primers were removed by adding 2.5U Exonuclease 1 (Exo, New England BioLab) and incubating at 37 °C for 30 min. Finally, the enzymes were denatured by further incubation at 80 °C for 15 min.

The PCR amplicons were subjected to dideoxy sequencing on an ABI 3730 DNA analyzer (Perkin Elmer). Sequencing was done in the forward and reverse direction using 27F and 1492R primers, respectively [34]. An additional primer, Com1 CAGCAGCCGCGGTAATAC, was used as an internal primer following the study of Schwieger and Tebbe [35]. The resulting ABI electrophoretograms were edited and assembled in Geneious 9.1.8.

### Identification and phylogenetic analysis of sequences

For identification, assembled sequences in FASTA format were individually queried in the GenBank and EZBioCloud databases and the resulting closest matching sequences were downloaded and used to build a multiple sequence alignment. Phylogenetic analysis was performed in mega version 11.0.13 [36]. FASTA sequences of actinomycete isolates were imported into mega and aligned using the muscle algorithm. The aligned sequences were inspected thoroughly, trimmed manually and realigned. The edited alignment was saved as a FASTA file and used to construct a phylogenetic tree using parameters of the best fitting model determined using the model function in mega. The resulting tree was exported in Newick format and stylized using iTOL version 6.7 [37].

## Results

Eight *Squamellaria* plants were sampled during this study, including three obligate *Squamellaria imberbis* plants associated with *Philidris nagasau*, one facultative *Squamellaria tenuiflora* plant associated with *Tetramorium insolens,* and four facultative *Squamellaria tenuiflora* plants inhabited by *Technomyrmex vitiensis* (Fig. 1).

### Distribution of actinomycetes in *Squamellaria* and associated ants

Actinomycetes were present in all three ant–plant mutualisms. *Squamellaria* samples generally yielded higher actinomycete colony forming units (CFUs) than ant cuticle and tissue samples. Actinomycetes were obtained from all samples with the exception of cuticle samples of *P. nagasau* and *T. insolens* (Table 1). However, in all cases, colonies originating from ant tissue and cuticle samples were too few to count and thus unsuitable for CFU estimation (Table S1). The highest actinomycete CFUs in this study were obtained from *S. imberbis* samples. Of the two isolation media used for *Squamellaria* samples, AIA produced more actinomycete CFUs than HVA for *S. imberbis* samples while HVA yielded more CFUs for *S*. *tenuiflora* samples (Table S1). Furthermore, *S. imberbis* samples displayed little to no actinomycete diversity and in most cases were found to be dominated by one actinomycete species (Fig. S1). In contrast, there were no clear dominant species in samples obtained from *S. tenuiflora* (Fig. S2).

**Table 1.** Sampled ant-plant mutualisms and respective number of actinomycete isolates obtained

Plant-ant mutualism	Colonies sampled	Number of actinomycete isolates			Total isolates
		Cuticle	Tissue	Nest	
*S. imberbis*-*P. nagasau*	3	0	1	5	6
*S. tenuiflora*-*Technomyrmex vitiensis*	4	5	9	15	29
*S. tenuiflora*-*Tetramorium insolens*	1	0	6	6	12
**Total**	**8**	**5**	**16**	**26**	**47**

**Fig. 2.**
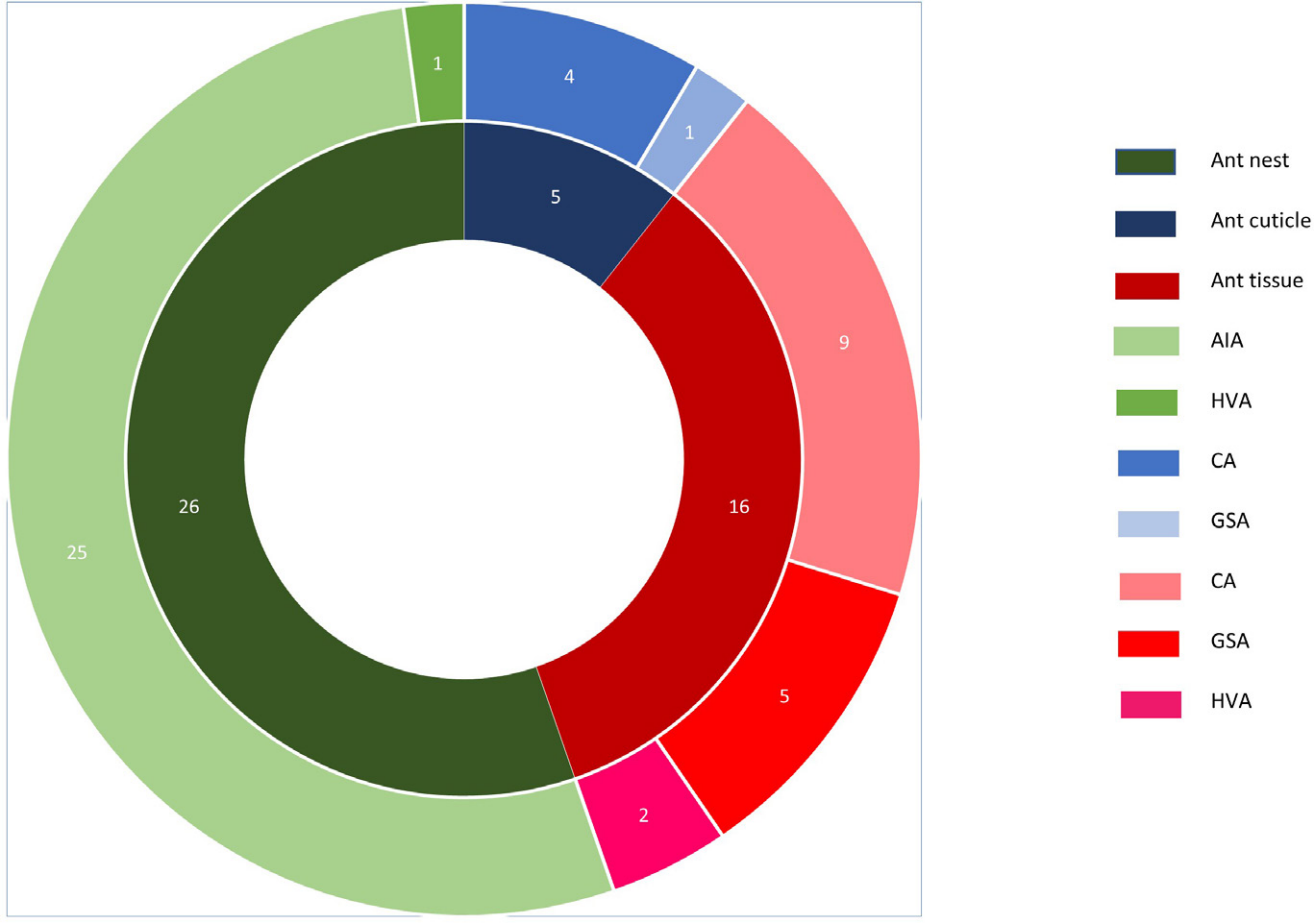
Distribution of isolates on samples and isolation media. AIA=actinomycetes isolation agar, HVA=humic acid vitamin agar, CA=chitin agar, and GSA=Gause’s synthetic agar.

A total of 47 actinomycetes were isolated from the samples including 6 from *P. nagasau,* 12 from *T. insolens* and 29 from *T. vitiensis* (Table 1). In all three studied species, cuticle samples yielded the lowest number of isolates while nest samples yielded the highest number (Table 1). The highest number of isolates were obtained from the *S. tenuiflora–T. vitiensis* mutualism. Considering isolation media, CA yielded the highest number of isolates (13) among the media used for ant cuticle and tissue samples (Fig. 2). On the other hand, for *Squamellaria* samples, the highest number of isolates were obtained on AIA (Fig. 2).

### Antibacterial activity of *Squamellaria* and ant-associated actinomycetes

Out of 47 actinomycetes isolated in this study, 12 displayed antibacterial activity against at least one out of the seven human pathogens tested. All antibacterial isolates originated from facultative *Squamellaria* and their respective ant associates and although the *P. nagasau*-associated *Squamellaria* yielded six isolates, none of the isolates displayed antibacterial activity against the tested pathogens.

Cross-streaking tests revealed that *E. coli* and *S. aureus* were the most susceptible test pathogens to our isolates, with each being inhibited by 5 isolates (Table 2). On the other hand, *S. enterica* was the most resistant pathogen as it was only susceptible to one isolate. Isolate TFS2010 displayed the broadest spectrum of activity, inhibiting 5 out of 7 test pathogens. Seven isolates displayed antibacterial activity on ISP2 agar, whereas six isolates showed antibacterial activity on MHA, and three isolates showed antibacterial activity on NA. Furthermore, 5 and 3 isolates displayed antibacterial activity exclusively on ISP2A and MHA, respectively. None of the isolates displayed antibacterial activity exclusively on NA plates.

**Table 2.** Cross-streaking results of 12 antibacterial isolates

Isolate	Growth medium	Test pathogens						
		* Enterococcus faecalis* ATCC 19433	* Staphylococcus aureus* ATCC 25923	* Escherichia coli* ATCC 25922	* Pseudomonas aeruginosa* ATCC 25668	* Klebsiella aerogenes *	* Salmonella enterica* NCTC 7836	* Shigella sonnei* ATCC 9290
**APS1004**	na	−	−	−	−	−	−	−
	MHA	−	−	−	+	−	−	−
	ISP2A	+	−	−	−	−	−	+
**APS1007**	na	−	−	−	−	−	−	−
	MHA	−	−	++	−	−	−	+
	ISP2A	−	−	−	−	−	−	−
**APS1011**	na	−	−	−	−	−	−	−
	MHA	−	+++	−	−	−	−	−
	ISP2A	−	−	−	−	−	−	−
**APS1012**	na	−	−	−	−	−	−	−
	MHA	−	−	−	−	−	−	−
	ISP2A	++	+++	−	−	−	−	−
**TFS1004**	na	−	−	−	−	−	−	−
	MHA	−	−	−	−	−	−	−
	ISP2A	+++	++	−	−	−	−	−
**TFS1005**	na	−	−	−	−	−	−	−
	MHA	−	−	−	−	−	−	−
	ISP2A	−	−	−	−	−	−	++
**TFS2003**	na	−	−	++++	−	++++	++++	−
	MHA	−	−	−	−	−	−	−
	ISP2A	−	−	−	−	−	−	−
**TFS2005**	na	−	−	−	−	−	−	−
	MHA	−	−	−	−	−	−	−
	ISP2A	−	−	+	−	−	−	−
**TFS2006**	na	−	+	−	−	−	−	−
	MHA	−	+	−	−	−	−	−
	ISP2A	−	−	−	−	−	−	−
**TFS2010**	na	++++	++++	−	−	++	−	−
	MHA	+++	+++	−	−	−	−	−
	ISP2A	++++	+++	+	+	++	−	−
**TFS3003**	na	−	−	−	−	−	−	−
	MHA	−	−	−	−	−	−	−
	ISP2A	−	−	+	−	−	−	−
**TFS4002**	na	−	−	−	−	−	−	−
	MHA	−	−	−	−	−	−	++
	ISP2A	−	−	−	−	−	−	−

+, ++, +++, ++++, ++++++, ++, +++, ++++, +++++ represent inhibition zone diameter ranges of 1–9 mm, 10–19 mm, 20–29 mm, 30–39 mm, and 40 and 49 mm, respectively. – represents a null inhibition zone diameter. NA – Nutrient agar, MHA – Mueller–Hinton agar, ISP2A – International Streptomyces Project 2 agar.

**Fig. 3.**
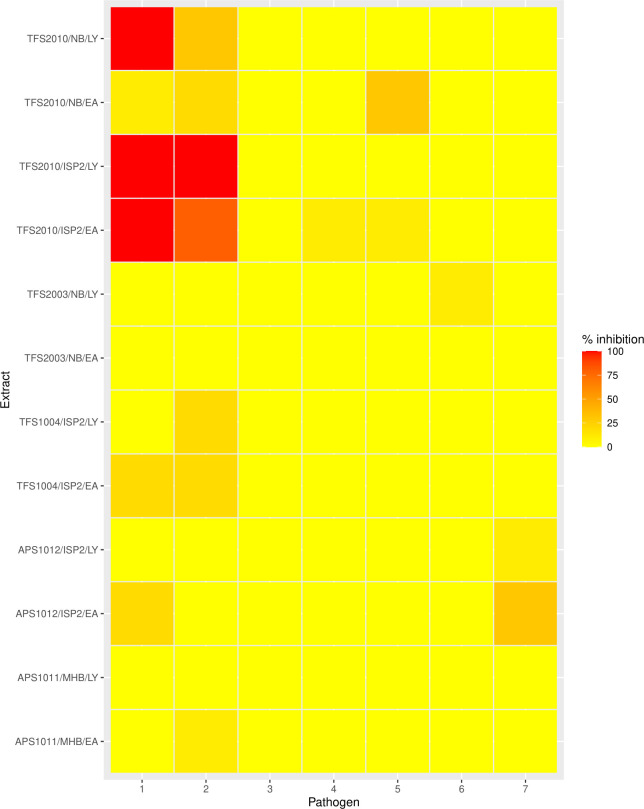
Heatmap showing activity of actinomycete crude extracts tested within the range of 25–0.049 mg ml^−1^ (0–100 %). NB=Nutrient broth, MHB=Mueller Hinton broth, EA=Ethyl acetate, LY=Lyophilized, 1= *Enterococcus faecalis* ATCC 19433, 2= *Staphylococcus aureus* ATCC 25923, 3=*Escherichia* coli ATCC 25922, 4= *Pseudomonas aeruginosa* ATCC 25668, 5= *Klebsiella aerogenes *, 6= *Salmonella enterica* NCTC 7836, and 7= *Shigella sonnei* ATCC 9290.

Based on primary screening results, five isolates including APS1011, APS1012, TFS1004, TFS2003 and TFS2010 were selected for batch cultivation, secondary metabolite extraction, and further screening. APS1011 was grown in MHB while TFS2003 was grown in Nutrient broth (NB). APS1012 and TFS1004 were grown in ISP2 broth (ISP2B). TFS2010 was cultivated in two broth media (i.e. ISP2B and NB) due to differences in antibacterial profile observed between the two media. Ethyl acetate and lyophilized extracts generally showed comparable inhibitory activity except in the case of TFS2010 (grown in NB) where the lyophilized extract displayed strong inhibitory activity (100 % inhibition) against *E. faecalis* while the ethyl acetate extract displayed weak inhibitory activity against the same pathogen (Fig. 3). Nonetheless, the most potent extracts were the ethyl acetate and lyophilized extracts of TFS2010 (grown in ISP2B), with the latter showing stronger inhibitory activity against *S. aureus *.

### Diversity and taxonomy of actinomycetes from *Squamellaria*–ant mutualisms

Nineteen isolates from our study including isolates that displayed good antibacterial activity in primary screening and all isolates derived from obligate *Squamellaria* samples were subjected to DNA extraction and 16S rRNA gene sequencing using 27F and 1492R primers. Searching the sequences in the EZBioCloud and GenBank databases revealed that our isolates’ closest matches belong to six actinomycete genera including *Amycolatopsis *, *Asanoa *, *Jiangella *, *Nocardia *, *Nocardiopsis,* and *Streptomyces *. About 68 % of isolates (15/21) matched to members of the genus *Streptomyces* with >98 % similarity in at least one of two databases (EZBioCloud and GenBank). Furthermore, approximately 10 % of isolates (i.e. 2/21) matched to *Asanoa siamensis* strain PS7-2. One isolate, PNS3004, matched to *Jiangella anatolica* and *J. asiatica* with 97.54 and 98.25% similarity in the EZBioCloud and GenBank databases and thus, is likely a novel species in the *Jiangella* genus (Table S2). PNS3004 matched, almost completely (99.85 %), to *Nocardiopsis dassonvillei* strain D1 in the EZBioCloud database (Tables S2 and S3). The percentage similarities of four isolates (i.e., TFS2015, TFS3001, PNS3002 and PNS3005) fell below 98.7 %, which has been proposed as the cutoff for the description of new species [38]. TFS2015 and TFS3001, which matched to members of the genera *Nocardia* and *Amycolatopsis,* respectively, exhibited the lowest percentage similarities to their corresponding type strains with percentage similarities below 96 % in both databases.

**Fig. 4.**
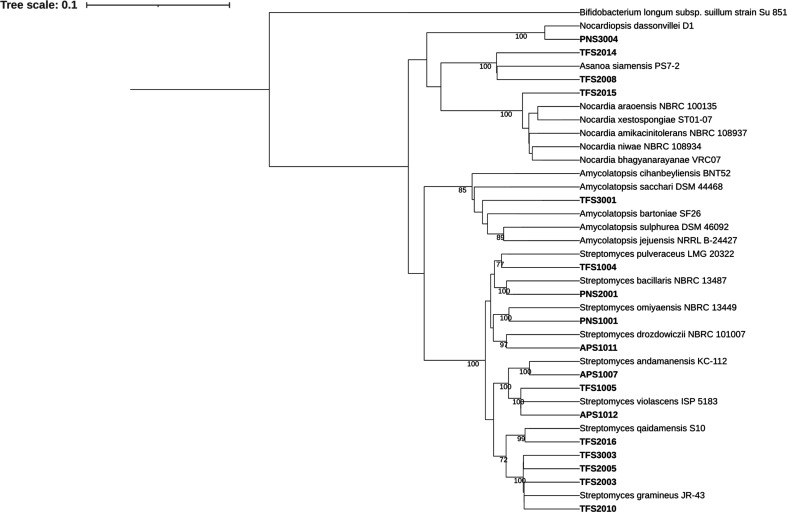
Neighbor-joining tree of 16S rRNA gene sequences of 17 isolates and their closest matches in the EZBioCloud database with *Bifidobacterium longum* as the outgroup. Numbers at nodes are bootstrap values (percentages of 1000 replications); only values >70 % are shown.

The *Streptomyces* isolates from our study formed two main clades, I and II. Clade I consists of *S. gramineus *, *S. qaidamensis*, *S. andamanensis* and *S. violascens* and closely related strains (Fig. 4). On the other hand, clade II comprises of *S. drozdowiczii *, *S. omiyaensis *, *S. bacillaris* and *S. pulveraceus* and related strains (Fig. 4). TFS3001 grouped with members of the genus *Amycolatopsis,* with *A. cihanbeyliensis* being the outgroup. PNS3004 grouped closely with *Nocardiopsis dassonvillei* while TFS2008 and TFS2014 grouped together with *Asanoa siamensis* (Fig. 4). TFS2015 shares the same common ancestor with *Nocardia* spp. but based on its positioning and low percentage similarity to its closest matches in two databases, may represent a new actinomycete genus.

## Discussion

In this study, we employed a strategy of isolation media diversification in order to improve the recovery of actinomycetes from samples. CA, GSA and HVA were used for the isolation of actinomycetes from ant tissue and cuticle samples while AIA and HVA were used for isolation from ant nest (i.e. *Squamellaria* tissue) samples. According to Baranova *et al*. [39], CA and GSA are among the most utilized isolation media for the isolation of actinomycetes from insect samples. Similarly, AIA and HVA are commonly used in the isolation of actinomycetes from environmental samples. Actinomycete colony counts obtained from tissue and cuticle samples were too low for CFU estimation (Table S1). However, for nest samples, AIA generally yielded higher CFU than HVA (Table S1). Actinomycetes were isolated from all three *Squamelleria*–ant associations and from all sampled components apart from cuticle samples of *P. nagasau* and *T. insolens*.

A total of 47 actinomycetes were isolated from 8 ant colonies, representing a yield of about 5.87 actinomycetes per colony (Table 1). More specifically, *P. nagasau–S. imberbis*, *T. vitiensis–S. tenuiflora* and *T. insolens–S. tenuiflora* samples yielded 2, 7.25 and 12 actinomycetes per colony, respectively (Table 1). In contrast, Hanshew *et al*. [16] reported a yield of 0.625(60/96) from *Pseudomyrmex penetrator–Tachigali* sp., 1.05(104/99) from *Petalomyrmex phylax–Leonardoxa africana*, and 3.025(121/40) from *Crematogaster margaritae–Keetia hispida* mutualisms. The differences in yield between studies could result from differences in host lifestyle and/or variations in actinomycete abundance in the host’s environment. Additionally, sampling strategy and isolation procedure are bound to affect actinomycete yield. In this study, ant nest samples yielded the highest number of actinomycete isolates (26 isolates) while cuticle samples yielded the least isolates (5 isolates) (Fig. 2). Most isolates were obtained from AIA plates while HVA yielded the least number of isolates for cuticle (0), tissue (2), and nest (1) samples (Fig. 2).

**Fig. 5.**
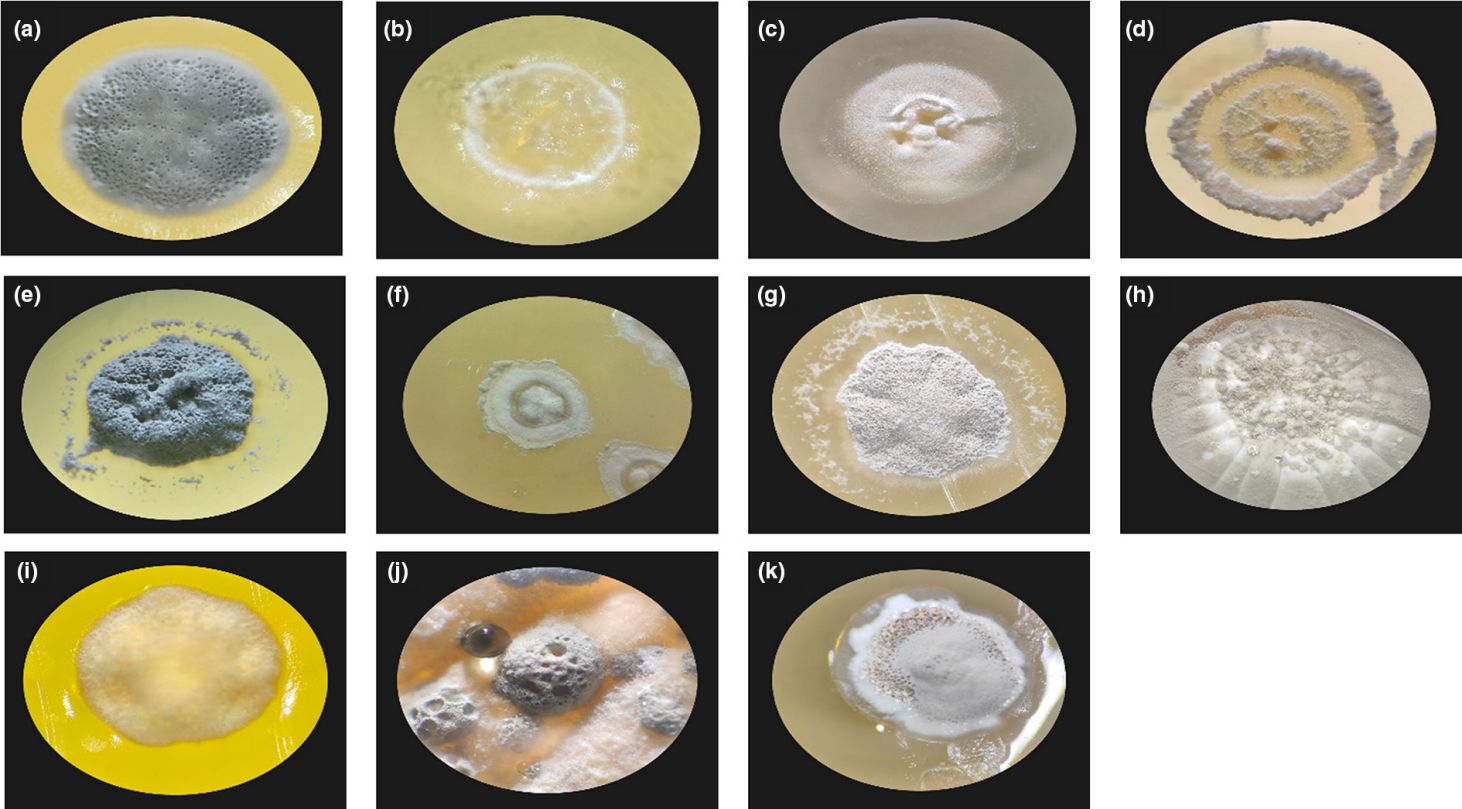
Light microscope images of actinomycete strains with antibacterial activity. (a) AP1004, (b) APS1007, (c) APS1011, (d) APS1012, (e) TFS1004, (f) TFS1005, (g) TFS2003, (h) TFS2005, (i) TFS2010, (j) TFS3003, (k) TFS4002. Image not available for strain TFS2006.

Cross-streaking tests revealed that our bioactive isolates are capable of inhibiting Gram-positive and Gram-negative pathogens alike, with *S. aureus* and *E. coli* being the most frequently inhibited (Fig. 5, Table 2). The least frequently inhibited pathogen was *S. enterica *, with only a single isolate exhibiting inhibitory activity towards it. Among the three media used for primary screening, ISP2A was the most suitable for secondary metabolism as more isolates exhibited antibacterial activity when grown on the medium (Table 2). In agreement with our observations, Rashad *et al*. [40] conducted bioactivity screening of actinomycetes on nine different solid media and found that the incidence of inhibitory activity was highest on ISP2A. TFS2003 and TFS2010 were the most bioactive actinomycetes in the cross-streaking tests, with both producing inhibition zones above 30 mm against two or more pathogens (Table 2). Incidentally, both isolates match to *Streptomyces gramineus* JR-43 with high percentage similarities>99 %. Strain JR-43 was originally isolated from the rhizosphere soil of Bamboo (*Sasa borealis*) collected in Jeonam, South Korea and has been shown to inhibit *Xanthomonas campestris* and *Xanthomonas axonopodis in vitro* (Tables S2 and S3). Nonetheless, the extracts of TFS2003 and TFS2010 differed significantly in antibacterial activity. Both extracts of TFS2010 (i.e. ethyl acetate and lyophilized) displayed potent antibacterial activity against selected pathogens (Fig. 3). In comparison, the lyophilized extract of TFS2003 displayed weak antibacterial activity against *Salmonella enterica* while the ethyl acetate extract of the strain was inactive against the tested pathogens (Fig. 3). Additionally, the antibacterial activity of NB and ISP2B extracts of TFS2010 were supported by cross-streaking results and may suggest nutrient-regulated antibacterial production in the strain.

By matching 16SrRNA sequences of 19 strains from this study to online genetic databases, it was determined that the strain’s closest matches span six actinomycete genera, including *Amycolatopsis *, *Asanoa *, *Jiangella *, *Nocardia *, *Nocardiopsis,* and *Streptomyces. Streptomyces* was the most common genus with about 68 % of strains matching to members of the genus with over 98 % similarity (Table S2). Similarly, several studies have reported high percentages of *Streptomyces* isolates [27, 41]. Furthermore, four isolates from this study are likely to belong to novel taxa. Strain TFS2015 displayed low match scores with a 95.44 % match to *Nocardia amikacinitolerans* NBRC 108937 in GenBank and 95.86 % match to *Nocardia* sp. WMMB213 in EZBioCloud (Table S2). Hatano *et al*. [42] reported the genus *Actinocrispum* with a percentage similarity of 95.7–96.2 % to members of the genus *Kibdelosporangium *. Additionally, phylogenetic analysis shows that TFS2015 is in a separate clade with its closest match, *Nocardia amikacinitolerans* NBRC 108937, and other closely related *Nocardia* spp. Thus, suggesting that TFS2015 may belong to a novel genus. Similarly, strain TFS3001 matched to *Amycolatopsis bartoniae* SF26 and *Amycolatopsis* sp. GM8 with 95.27 and 95.90%, respectively. However, since the strain grouped within the *Amycolatopsis* clade, it is likely a novel species within the genus. Two other strains including PNS3002 and PNS3005 exhibited percentage similarities below the threshold for new bacterial species (i.e. 98.7 %) and are likely to be new members of *Jiangella* and *Streptomyces,* respectively (Table S2).

Phylogenetic analysis of strains from this study revealed that the *Streptomyces* strains form two distinct clades. The first clade contains *S. gramineus *, *S. andamanensis *, *S. qaidamensis* and *S. violascens* and 8 closely related isolates from our study (Fig. 4). Four of the eight isolates in clade I were derived from the *S. tenuiflora–T. vitiensis* mutualism and displayed high similarity to *S. gramineus* (Table S3). In the second clade *S. drozdowiczii *, *S. omiyaensis *, *S. bacillaris* and *S. pulveraceus* group together with four of our isolates. Interestingly, PNS1001 and PNS2001, despite originating from two separate *Squamellaria* plants, are closely related species (Fig. 4, Table S2). The abundance and dominance of two closely related *Streptomyces* species in two separate *S. imberbis* samples may indicate that certain *Streptomyces* lineages provide specialized functions in the obligate *Squamellaria* mutualism. However, further sampling is needed to confirm this association since the third *Squamellaria imberbis* plant sampled in this study displayed an abundance of a *Jiangella* strain (i.e. PNS3002) rather than a *Streptomyces* sp. (Fig. S1). Furthermore, actinomycetes from the *S. imberbis–P*. *nagasau* association, unlike those derived from the facultative mutualisms, did not display inhibitory activity against the human pathogens tested (Table 2). Although our attempts to screen these isolates against the nest parasites of *P. nagasau* were unsuccessful, the closest matches of some of the isolates are known to improve plant fitness (Table S2).

## Conclusion

Actinomycetes were isolated from all three studied *Squamellaria*–ant associations with notable differences in abundance between obligate and facultative mutualisms. Although, most isolates belonged to the *Streptomyces* genus, five other genera were represented including *Amycolatopsis *, *Asanoa *, *Jiangella *, *Nocardia* and *Nocardiopsis *. Bioassay results show that several isolates exhibit antibacterial activity against human pathogenic bacteria and thus *Squamellaria*-ant associations may be a potential source of therapeutics. However, more work is needed especially in the characterization of the bioactive compounds produced by the actinomycete isolates. In summary, this is the first study to report actinomycetes from *Squamellaria-*ant mutualisms and adds to the growing body of work on actinomycetes from insect mutualisms.

## Availability of data and material

The datasets generated during and/or analysed during the current study are available from the corresponding author on reasonable request.

## Supplementary Data

Supplementary material 1
