# Supplementary material for: Isolation, characterization and screening of actinomycetes associated with fijian ant–plant symbioses
Source: Microbiology (Reading). 2023 Nov 8;169(11):001410. doi: 10.1099/mic.0.001410 (PMC10710841; doi:10.1099/mic.0.001410)
Supplement: Supplementary material 1 [file mic-169-1410-s001.pdf]

SUPPLEMENTARY TABLES

Supplementary table 1. Ant-plant sampling details

Hymenopteran species	Colony ID	Sampling location	Collection date	Actinomycete abundance (CFU/g)	
				AIA	HVA
Philidrisnagasau	PNS1	Near Waisali rainforest reserve, Savusavu (16°37'46.8"S 179°12'14.8"E)	25/07/2022	$10.9 \times 10^{4} \pm 4000.0$	$8.2 \times 10^{4} \pm 7000.0$
	PNS2	Near Waisali rainforest reserve, Savusavu (16°37'46.8"S 179°12'14.8"E)	25/07/2022	$2. 9 \times 10^{3} \pm 416.3$	$2. 3 \times 10^{3} \pm 152.8$
	PNS3	Near Waisali rainforest reserve, Savusavu (16°37'46.8"S 179°12'14.8"E)	25/07/2022	$5.7 \times 10^{3} \pm 264.6$	$9.1 \times 10^{5} \pm 75055.5$
Technomyrmexvitiensis	TFS1	Colo-i-Suva Forest Reserve (18° 3'38.65"S, 178°28'12.53"E)	13/12/2021	TFTC	TFTC
	TFS2	Colo-i-Suva Forest Reserve (18° 3'34.80"S, 178°28'15.69"E)	14/03/2022	TFTC	TFTC
	TFS3	Colo-i-Suva Forest Reserve (18° 3'14.37"S, 178°27'47.24"E)	28/03/2022	TFTC	TFTC
	TFS4	Colo-i-Suva Forest Reserve (18° 3'15.21"S, 178°27'48.00"E)	28/03/2022	TFTC	TFTC
Tetramoriuminsolens	APS1	Colo-i-Suva Forest Reserve (18° 3'16.05"S, 178°27'46.69"E)	17/11/2021	TFTC	TFTC

Actinomycete abundanceis expressed as Mean CFU ± Standard deviation. TFTC – Too few to count

Supplementary table 2. Isolates and their closest matches in two databases (EZBioCloud and GenBank)

Isolate	EZBioCloud			GenBank		
	**Top hit strain**	**Percentage similarity**	**Description and Biological activity**	**Top hit strain**	**Percentage similarity**	**Description and Biological activity**
APS1007	*Streptomycesandamanensis* KC-112	98.83	*Streptomycesandamanensis*was first isolated from soil collected in the Similan islands of Thailand (Sripreechasak et al., 2016). There are no reports of compounds produced by this species.	*Streptomyces* sp. SAT1	98.95	*Streptomyces* sp. SAT1 is a plant endophyte (Martín-Sánchez et al., 2019). AntiSMASH analysis of strain SAT1 genome showed that the strain contains several BGCs including those coding for Bafilomycin B1,Coelichelin, Albaflavenone, Flaviolin and several more unknown compounds.
APS1011	*Streptomyces drozdowiczii*NBRC 101007	99.41	The species was first isolated from a soil sample collected inMata Atlântica, Brazil. Strain M7a shows antimicrobial activity against *Rhizopus stolonifer* and *Escherichia coli*(Semêdo et al., 2004).	*Streptomyces drozdowiczii* DGA8-3	99.18	Similar to EZBioCloud hit.
APS1012	*Streptomyces albidoflavus*DSM 40455	99.41	*S. albidoflavus* was first isolated from a soil sample collected in Poland and is the producer of the antibacterial Streptothricin. A strain isolated from *Camponotusvagus*(Carpenter ant) was found to inhibit *Candida albicans* in vitro and produce Antimycin A (Baranova et al., 2020).	*Streptomyces lividans*N309	98.95	*S. lividans* was originally isolated from. Like *S. coelicolor*, the species is known to produce the well-known antibiotics Actinorhodin, Calcium-dependent antibiotic, and Undecylprodigiosin.

PNS1001	*Streptomyces omiyaensis*NBRC 13449	99.88	*S. omiyaensis*was isolated from soil in Omiya, Japan. AntiSMASH analysis of strain JCM4806 shows BGCs for Chloramphenicol, Desferrioxamin B, Venezuelin and several other natural products.	*Streptomyces venezuelae* ATCC 10712	99.88	Originally isolated from soil collected in Venezuela, this species is best known for producingChloramphenicol, Jadomycin and Pikromycin. Furthermore, Yoolong et al., (2019)demonstrated that *Streptomyces venezuelae*ATCC 10712 enhances plant fitness.
PNS2001	*Streptomyces bacillaris*NBRC 13487	99.38	*Streptomyces bacillaris* was originally isolated from forest soil collected in Oregon, USA and is known to produce plant growth promoting compounds such as ACC deaminase. The species is also known to fix nitrogen and mobilize phytate (Cardinale et al., 2015).	*Streptomyces bacillaris*S_991	99.13	Similar to EZBioCloud hit.
PNS3002	*Jiangellaanatolica* GTF31	97.54	StrainGTF31 was isolated from a lake soil sample from Anatolia, Turkey (Ay et al., 2019). Analysis of BGCs of the strain revealed that it contains a BGC for alkylresorcinol and several other unknown natural products.	*Jiangella asiatica* 5K138	98.25	*Jiangella asiatica* was first isolated from Soil from the Karakum Desert of Turkmenistan (Saygin et al., 2020). Like*J. anatolica*, the species contains a BGC for alkylresorcinol. In addition, strain 5K138 contains BGCs for a lanthipeptide (60% similarity in antiSMASH)
PNS3004	*Nocardiopsisdassonvillei* subsp. *crassaminis* D1	99.85	*N. dassonvillei* was first isolated from mildewed grain(Beau et al., 1999). Fu et al., (2011) reported three new α-pyrones (Nocapyrones E−G) and three newthree new diketopiperazine derivatives (Nocazines A−C) from	*Nocardiopsis* sp. YGBL-10	99.85	Unknown

			strain HR10-5. The former compounds were shown to exhibit antibacterial activity against *Bacillus subtilis*.			
PNS3005	*Streptomyces siamensis*KC038	98.70	This species was first isolated from a soil sample collected in Thailand. There are no reports of bioactivity from the species	*Streptomyces* sp.BV9	97.53	
TFS1004	*Streptomyces pulveraceus*LMG 20322	99.25	This species was first isolated from a soil sample in Fukuchiyama, Japan and was found to produce two metabolites with wide ranging antimicrobial properties,Zygomycin A and B(Shibata et al., 1961). Furthermore, a family of polycyclic tetramate macrolactams was detected in the culture broth of strain ES16 which was isolated from the root of an apple tree. Other secondary metabolites produced by the species include Epiderstatin, Actiketal, and Fostriecin(Sonoda et al., 1991).	*Streptomyces pulveraceus* MJM11727	99.75	Similar to EZBioCloud hit.
TFS1005	*Streptomyces violascens* ISP 5183	99.33	The antibacterials Albaflavenone(Zheng et al., 2016), Violapyrones A–G(Zhang et al., 2020) and Cyclo(phe-pro)(Viswapriya& Saravana Kumari, 2022) are produced by the species.	*Streptomyces* sp.HBUM206360	99.67	Unknown

TFS2003	*Streptomyces gramineus*JR-43	99.40	Strain JR-43 was isolated from the rhizosphere of soil of Bamboo (*Sasa borealis*). The strain is known to inhibit the plant pathogens *Xanthomonas campestris* and *Xanthomonas axonopodis*(Lee et al., 2012). Ma et al., (2018) isolated six novel compounds, Actinofuranones D-I, from a *S. gramineus*strains (YIM 130461) isolated from lichen.	*Streptomyces gramineus* S3	99.76	Similar to EZBioCloud hit.
TFS2005	*Streptomyces gramineus*JR-43	99.87	Similar to TFS2003	*Streptomyces* sp. GF24	99.61	Unknown
TFS2010	*Streptomyces gramineus*JR-43	99.40	Similar to TFS2003	*Streptomyces gramineus* S3	99.76	Similar to TFS2003
TFS2011	*Asanoasiamensis* PS7-2	99.27	*Asanoasiamensis*was originally isolated from peat swamp forest soil in Thailand (Niemhom et al., 2013). AntiSMASH analysis revealed that strain NBRC 107932 contains BGCs for terpenes, lanthipepetides, and non-ribosomal polyketide synthases (NRPS).	*Asanoasiamensis* PS7-2	98.70	Similar to EZBioCloud hit
TFS2014	*Asanoasiamensis* PS7-2	98.98	Similar to TFS2011	*Asanoasiamensis* PS7-2	98.70	Similar to TFS2011

TFS2015	*Nocardia amikacinitolerans*NBRC 108937	95.44	This species was first isolated from a human patient and was found to exhibit resistance to Amikacin (Ezeoke et al., 2013). AntiSMASH analysis of the whole genome of strain DSM 45539 showed that the strain contains BGCs for several NRPSs and terpenes.	*Nocardia* sp. WMMB213	95.86	Unknown
TFS2016	*Streptomyces qaidamensis* S10	99.53	Strain S10T, was isolated from a sand sample collected from the Qaidam Basin in China(B. Zhang et al., 2018). The strain exhibits antibacterial activity against Methicillin resistant Staphylococcus aureus (MRSA)	*Streptomyces pseudovenezuelae* SKH1-3	99.84	Originally isolated from lead polluted soil in China, *Streptomyces pseudovenezuelae*is a producerof Chloramphenicol and the rare tetrahydroanthracene antibiotic, Setomimycin.
TFS3001	*Amycolatopsisbartoniae* SF26	95.27	*Amycolatopsisbartoniae* strain SF26 was isolated from sandy soil sample collected in Australia in 2012 (Zucchi et al., 2012). The genome of strain CGMCC 4.7679 contains BGCs for NRPSs, polyketides and lanthipeptides.	*Amycolatopsis* sp. GM8	95.90	Unknown
TFS3003	*Streptomyces gramineus* JR-43	99.63	Similar to TFS2003	*Streptomyces* sp. Mi_A_43	99.38	Unknown

Supplementary table 3 Appearanceof isolates and their closest matches

**Isolate**	**EZBioCloud match**	**GenBank match**
APS1007 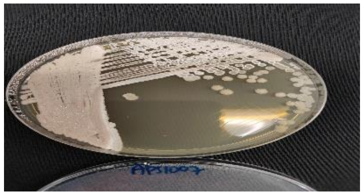	*Streptomycesandamanensis* KC-112	*Streptomyces* sp. SAT1
APS1011 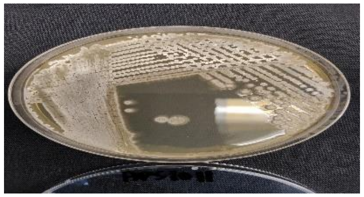	*Streptomyces drozdowiczii*NBRC 101007	*Streptomyces drozdowiczii* DGA8-3 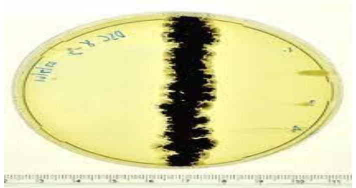 (Promnuan et al., 2020)

APS1012 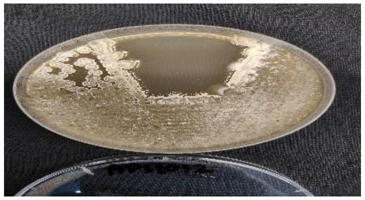	*Streptomyces albidoflavus*DSM 40455 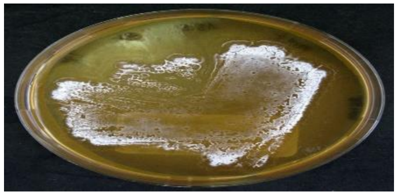 (https://bacdive.dsmz.de/strain/14904)	*Streptomyces lividans*N309
PNS1001 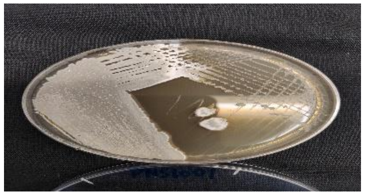	*Streptomyces omiyaensis*NBRC 13449	*Streptomyces venezuelae* ATCC 10712 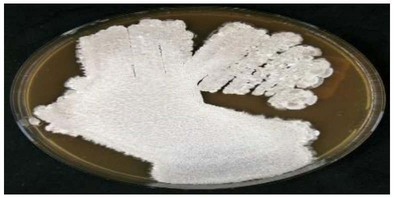
PNS2001 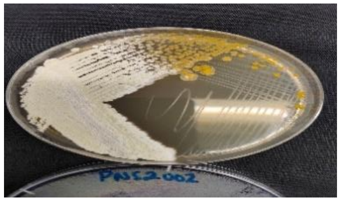	*Streptomyces bacillaris*NBRC 13487 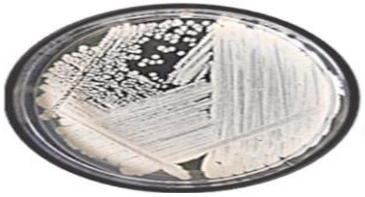	*Streptomyces bacillaris*S_991
PNS3002 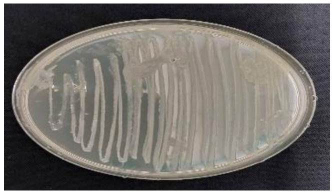	*Jiangellaanatolica* GTF31	*Jiangella asiatica* 5K138

PNS3004 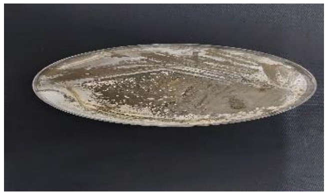	*Nocardiopsisdassonvillei* subsp. *crassaminis* D1 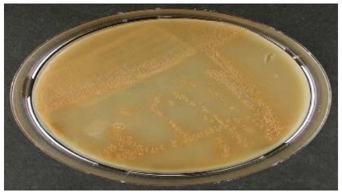	*Nocardiopsis* sp. YGBL-10
PNS3005 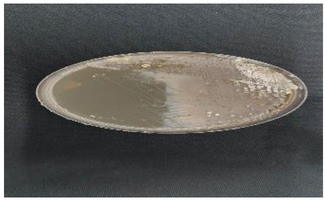	*Streptomyces siamensis*KC038	*Streptomyces* sp.BV9
TFS1004 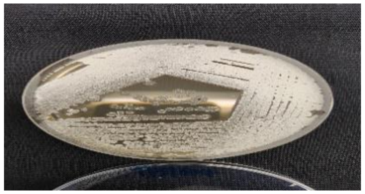	*Streptomyces pulveraceus*LMG 20322	*Streptomyces pulveraceus* MJM11727
TFS1005 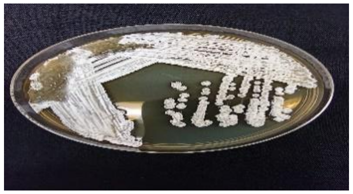	*Streptomyces violascens* ISP 5183	*Streptomyces* sp.HBUM206360

TFS2003<br>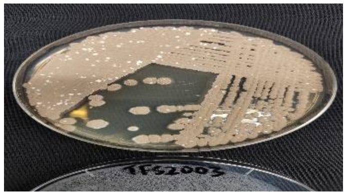	*Streptomyces gramineus*JR-43<br>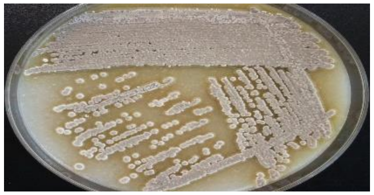<br>Contributor: H.J. Lee & K.S. Whang.(http://www.actino.jp/DigitalAtlas/index.html#tabs-use:~:text=(https%3A//atlas.actino.jp/)	*Streptomyces gramineus* S3
TFS2005<br>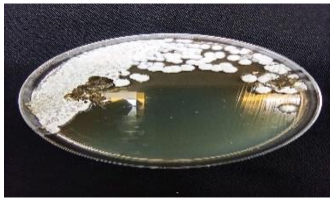	*Streptomyces gramineus*JR-43<br>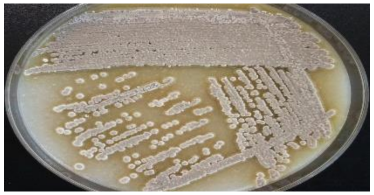	*Streptomyces* sp. GF24
TFS2010<br>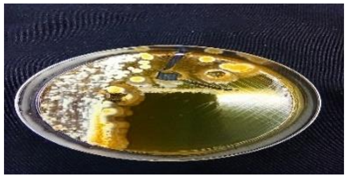	*Streptomyces gramineus*JR-43<br>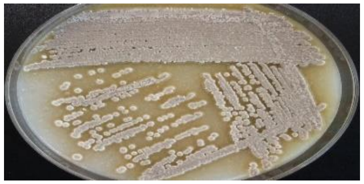	*Streptomyces gramineus* S3

TFS2011 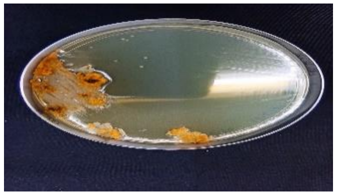	*Asanoasiamensis* PS7-2 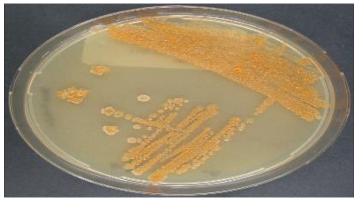 *https://bacdive.dsmz.de/strain/7853*	*Asanoasiamensis* PS7-2
TFS2014 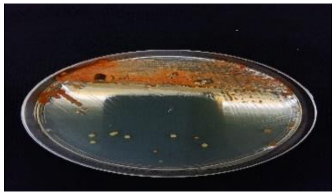	*Asanoasiamensis* PS7-2 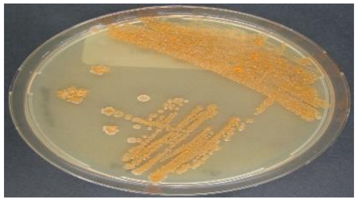	*Asanoasiamensis* PS7-2
TFS2015 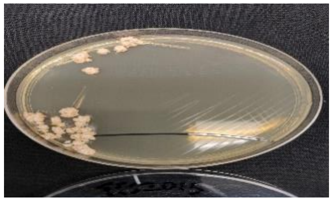	*Nocardia amikacinitolerans*NBRC 108937	*Nocardia* sp. WMMB213

TFS2016 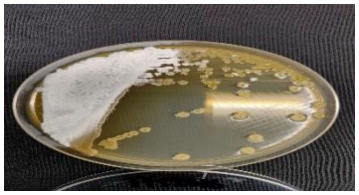	*Streptomyces qaidamensis* S10	*Streptomyces pseudovenezuelae* SKH1-3
TFS3001 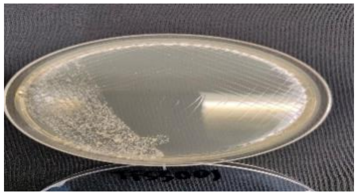	*Amycolatopsisbartoniae* SF26	*Amycolatopsis* sp. GM8
TFS3003 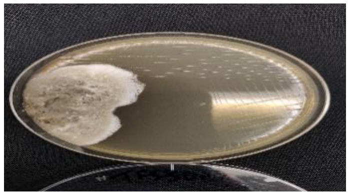	*Streptomyces gramineus* JR-43 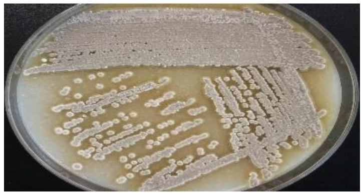	*Streptomyces* sp. Mi_A_43

SUPPLEMENTARY FIGURES

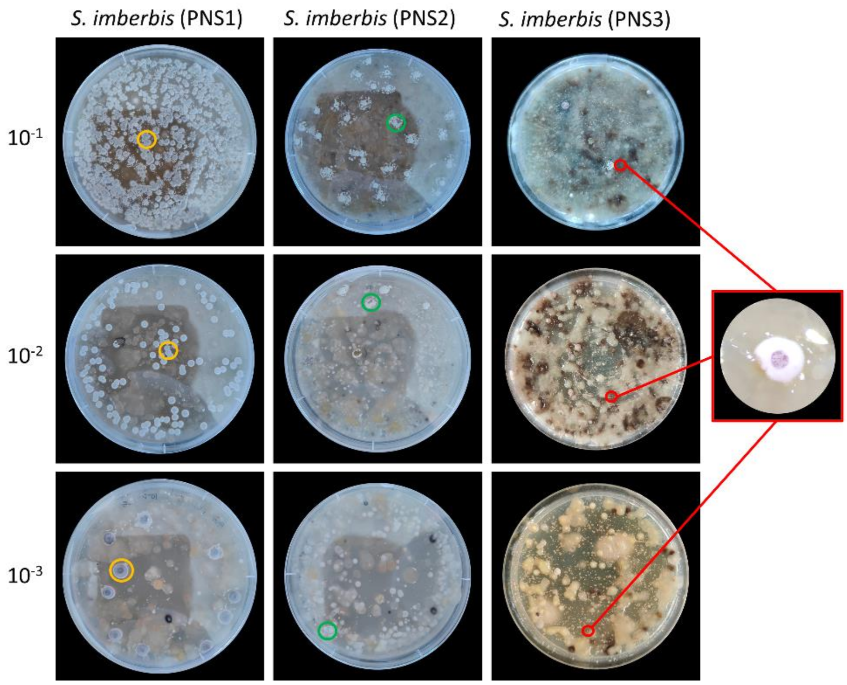

Supplementary figure 1. Photos of isolation plates spread with different dilutions of *S. imberbis* samples (10^{-1}, 10^{-2},10^{-3}) taken after 4 weeks incubation at 28 ^{o}C.Colored circles indicate dominant actinomycetes

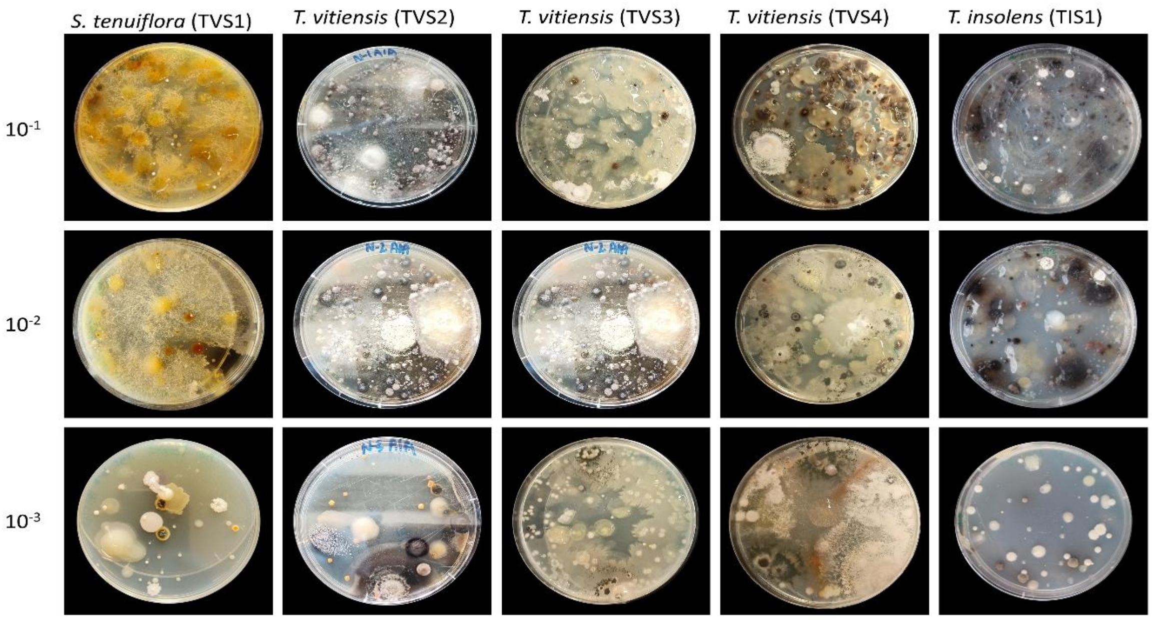

Supplementary figure 2. Photos of isolation plates spread with different dilutions of *S. tenuiflora*samples (10^{-1}, 10^{-2},10^{-3}) taken after 4 weeks incubation at 28 ^{o}C

REFERENCES

AyH, NouiouiI, CarroL, KlenkH-P, CetinD, et al. *Jiangellaanatolica* sp. nov. isolated from coastal lake soil. *Antonie van Leeuwenhoek*2019;112:887–895. 10.1007/s10482-018-01222-y

BaranovaAA, ChistovAA, TyurinAP, ProkhorenkoIA, KorshunVA, et al. Chemical ecology of *Streptomyces albidoflavus* strain A10 associated with carpenter ant *Camponotusvagus*. *Microorganisms*2020;8:1948. 10.3390/microorganisms8121948

BeauF, BolletC, CotonT, GarnotelE, DrancourtM. Molecular identification of a *Nocardiopsisdassonvillei* blood isolate. *J Clin Microbiol*1999;37:3366–3368. 10.1128/JCM.37.10.3366-3368.1999

CardinaleM, RateringS, SuarezC, Zapata MontoyaAM, Geissler-PlaumR, et al. Paradox of plant growth promotion potential of rhizobacteria and their actual promotion effect on growth of barley (*Hordeum vulgare* L.) under salt stress. *Microbiol Res*2015;181:22–32. 10.1016/j.micres.2015.08.002

EzeokeI, KlenkH-P, PötterG, SchumannP, MoserBD, et al. *Nocardia amikacinitolerans* sp. nov., an amikacin-resistant human pathogen. *Int J Syst EvolMicrobiol*2013;63:1056–1061. 10.1099/ijs.0.039990-0

Fu P, Liu P, Qu H, Wang Y, Chen D, et al. Α-pyrones and diketopiperazine derivatives from the marine-derived actinomycete *Nocardiopsisdassonvillei* HR10-5. *J Nat Prod* 2011;74:2219–2223. 10.1021/np200597m

LeeH-J, HanS-I, WhangK-S. *Streptomyces gramineus* sp. nov., an antibiotic-producing actinobacterium isolated from bamboo (*Sasa borealis*) rhizosphere soil. *Int J Syst EvolMicrobiol*2012;62:856–859. 10.1099/ijs.0.030163-0

MaJ, CaoB, LiuC, GuanP, MuY, et al. Actinofuranones D-I from a lichen-associated actinomycetes, *Streptomyces gramineus*, and their anti-inflammatory effects. *Molecules :J Syn Chem Nat Prod Chem*2018;23:2393. 10.3390/molecules23092393

Martín-SánchezL, SinghKS, AvalosM, van WezelGP, DickschatJS, et al. Phylogenomic analyses and distribution of terpene synthases among *Streptomyces*. *Beilstein J Org Chem*2019;15:1181–1193. 10.3762/bjoc.15.115

NiemhomN, SuriyachadkunC, TamuraT, ThawaiC. *Asanoasiamensis* sp. nov., isolated from soil from a temperate peat swamp forest. *Int J Syst EvolMicrobiol*2013;63:66–71. 10.1099/ijs.0.038851-0

PromnuanY, PromsaiS, MeelaiS. Antimicrobial activity of *Streptomyces* spp. isolated from *Apis dorsata* combs against some phytopathogenic bacteria. *PeerJ*2020;8:e10512. 10.7717/peerj.10512

SayginH, AyH, GuvenK, SahinN. Genome-based classification of three novel actinobacteria from the Karakum desert: *Jiangella asiatica* sp. nov., *Jiangellaaurantiaca* sp. nov. and *Jiangellaureilytica* sp. nov.*Int J Syst EvolMicrobiol*2020;70:1993–2002. 10.1099/ijsem.0.004011

SemêdoL, GomesRC, LinharesAA, DuarteGF, NascimentoRP, et al. *Streptomyces drozdowiczii* sp. nov., a novel cellulolytic streptomycete from soil in Brazil. *Int J Syst EvolMicrobiol*2004;54:1323–1328. 10.1099/ijs.0.02844-0

ShibataM, HigashideE, KanzakiT, YamamotoH, NakazawaK. Studies on Streptomycetes. *Agric Biol Chem*1961;25:171–199. 10.1080/00021369.1961.10857791

SonodaD, OsadaH, UzawaJ, IsonoK. Actiketal, a new member of the glutarimide antibiotics. *J Antibiot*1991;44:160–163. 10.7164/antibiotics.44.160

SripreechasakP, TamuraT, ShibataC, SuwanboriruxK, TanasupawatS. *Streptomyces andamanensis* sp. nov., isolated from soil. *Int J Syst EvolMicrobiol*2016;66:2030–2034. 10.1099/ijsem.0.000987

ViswapriyaV, Saravana KumariP. Synergistic activity of cyclo(phe-pro) antibiotic from *Streptomyces violascens* VS in reducing the drug resistance burden of clinically significant pathogens. *J Biol Act Prod Nat*2022;12:450–460. 10.1080/22311866.2022.2162579

YoolongS, KruasuwanW, Thanh PhạmHT, JaemsaengR, JantasuriyaratC, et al. Modulation of salt tolerance in Tthai jasmine rice (*Oryza sativa* L. cv. KDML105) by *Streptomyces venezuelae* ATCC 10712 expressing ACC deaminase. *Sci Rep*2019;9:1275. 10.1038/s41598-018-37987-5

ZhangB, TangS, ChenX, ZhangG, ZhangW, et al. *Streptomyces qaidamensis* sp. nov., isolated from sand in the Qaidam Basin, China. *J Antibiot*2018;71:880–886. 10.1038/s41429-018-0080-9

ZhangL, ZhangJ, RenB, LuW, HouC, et al. Characterization of anti-BCG benz[α]anthraquinones and new siderophores from a Xinjiang desert-isolated rare actinomycete *Nocardia* sp. XJ31. *Appl MicrobiolBiotechnol*2020;104:1–12. 10.1007/s00253-020-10842-2

ZhengD, DingN, JiangY, ZhangJ, MaJ, et al. Albaflavenoid, a new tricyclic sesquiterpenoid from *Streptomyces violascens*. *J Antibiot*2016;69:773–775. 10.1038/ja.2016.12

ZucchiTD, BondaANV, FrankS, KimB-Y, KshetrimayumJD, et al. *Amycolatopsisbartoniae* sp. nov. and *Amycolatopsisbullii* sp. nov., mesophilic actinomycetes isolated from arid Australian soils. *Antonie van Leeuwenhoek*2012;102:91–98. 10.1007/s10482-012-9716-0
